# An Indoor Fire Detection Method Based on Multi-Sensor Fusion and a Lightweight Convolutional Neural Network

**DOI:** 10.3390/s23249689

**Published:** 2023-12-07

**Authors:** Xinwei Deng, Xuewei Shi, Haosen Wang, Qianli Wang, Jun Bao, Zhuming Chen

**Affiliations:** 1 Yangtze Delta Region Institute (Quzhou), University of Electronic Science and Technology of China, Quzhou 324003, China; 202122011142@std.uestc.edu.cn (X.D.); 202221010509@std.uestc.edu.cn (X.S.); 2 School of Information and Communication Engineering, University of Electronic Science and Technology of China, Chengdu 611731, China; wangh8972@gmail.com (H.W.); baojun@uestc.edu.cn (J.B.); 3 School of Information Science and Technology, Southwest Jiaotong University, Chengdu 611756, China; qlwang@swjtu.edu.cn

**Keywords:** indoor fire detection, fire numerical simulation, sensor data fusion, time-series imaging, embedded platform

## Abstract

Indoor fires pose significant threats in terms of casualties and economic losses globally. Thus, it is vital to accurately detect indoor fires at an early stage. To improve the accuracy of indoor fire detection for the resource-constrained embedded platform, an indoor fire detection method based on multi-sensor fusion and a lightweight convolutional neural network (CNN) is proposed. Firstly, the Savitzky–Golay (SG) filter is used to clean the three types of heterogeneous sensor data, then the cleaned sensor data are transformed by means of the Gramian Angular Field (GAF) method into matrices, which are finally integrated into a three-dimensional matrix. This preprocessing stage will preserve temporal dependency and enlarge the characteristics of time-series data. Therefore, we could reduce the number of blocks, channels and layers in the network, leading to a lightweight CNN for indoor fire detection. Furthermore, we use the Fire Dynamic Simulator (FDS) to simulate data for the training stage, enhancing the robustness of the network. The fire detection performance of the proposed method is verified through an experiment. It was found that the proposed method achieved an impressive accuracy of 99.1%, while the number of CNN parameters and the amount of computation is still small, which is more suitable for the resource-constrained embedded platform of an indoor fire detection system.

## 1. Introduction

Fire is one of the disasters that pose a great threat to the safety of people’s lives and property. Statistics provided by the International Association of Fire and Rescue Services (CTIF) in 2022 show that there have been 104 million fires across 50 countries in the past 27 years, most of which are indoor fires [1]. The total number of deaths in those 27 years is 1.1236 million. If an indoor fire is detected at an early stage, a lot of people might be saved. Therefore, indoor fire detection is indeed a vital concern that requires effective solutions and technologies to mitigate the devastating consequences associated with such incidents.

Early fire detection systems have relied on a single temperature sensor or smoke sensor, employing a single threshold for judgment. However, these systems are susceptible to interference and often exhibit low detection accuracy. The corresponding fire detection methods include the threshold method, the trend method [2], the power spectrum algorithm [3], and so on. While these approaches are straightforward to implement, their accuracy is limited. To address these limitations, composite detectors are widely used nowadays [4]. Some scholars collect smoke concentration, temperature, carbon monoxide (CO) concentration, images and other information, and apply information fusion technology to fuse the heterogeneous data of multi-sensors [5,6]. Their detection accuracy is much higher than that of the early systems, and the anti-interference ability is stronger. In addition, the use of wireless sensors [7,8] connected to the Internet enables faster response and wider coverage of the detection system.

The prevailing multi-sensor data fusion methods for fire detection can be divided into two categories. The first category is based on statistics and inference [9]. The second category is based on neural networks.

Examples of the first type of method: Wang et al. [10] proposed a fire detection system based on the Kalman filter. By employing the Kalman filter, they effectively integrated sensor data and generated outputs indicating the probabilities of no-fire, flaming, and smoldering within the system. Rachman et al. [11] employed fuzzy logic rules to fuse data from various sensors in complex fire scenarios. Wang et al. [12] designed and performed modified hierarchical analysis to determine the weight of each sensor, subsequently utilizing the multivariate weighted fusion method to assess the probability of fire occurrence. Maltezos et al. [13] used edge computing technology to overcome the shortcomings of the fire perception system based on the Internet of Things, such as limited energy resources and a lack of real-time computer processing ability. These methods improve the real-time accuracy of the fire detection system to a certain extent, but the accuracy is not satisfactory.

Examples of the second type of method: GuoPing Jiang et al. [14] presented an approach wherein information concerning smoke concentration, CO concentration, and temperature is fused, and an improved Back Propagation Neural Network (BPNN) is proposed to classify the fire scene. Similarly, Deng et al. [15] employed an adaptive weight adjustment technique to combine a BPNN, resulting in commendable performance. These methods addressed the problem of BP neural networks tending to fall to local minima during the training process. However, the BPNN still faces certain issues, such as a poor fitting effect, susceptibility to noise, and slow training speed on large datasets. Therefore, some scholars have tried to use more complex networks to achieve data fusion, such as probabilistic neural networks [16] or wavelet neural networks [17]. Baek et al. [18] used a dynamic time-warping (DTW) algorithm to assess the similarity of sensor data before and after a fire. Furthermore, they proposed a k-out-of-p rule based on p-channel sensor data to make adaptive decisions. Sun et al. [19] leveraged LSTM to extract features from multi-sensor fusion and output the probabilities of no-fire, flaming, and smoldering. Moreover, a decision tree algorithm was employed to yield the final fire detection outcome. With the development of deep learning, the Convolutional Neural Network (CNN) has been proposed and applied to fire detection. Using a CNN to detect fire based on video frames can achieve remarkable accuracy, but comes with substantial computational complexity. Consequently, it is difficult to implement in a resource-constrained embedded platform.

Therefore, in this paper, we aim to use the Savitzky–Golay (SG) filter and the Gramian Angular Field (GAF) method to pre-process the heterogeneous sensor data, i.e., the smoke, CO, and temperature sensor data, and construct the three-dimensional matrices. The SG filter can be regarded as a de-noising procedure, and the GAF method can effectively preserve temporal dependency and enlarge the characteristics of the time-series information. Then, the improved ConvNeXt structure proposed by Zhuang Liu et al. [20] is used for fire detection due to its advantages of simplicity and efficiency, leading to a lightweight CNN named ConvNeXt-FiRe for indoor fire detection, while keeping an impressive level of accuracy.

The rest of this article is organized as follows: The introduction and analysis of the indoor fire detection method are in Section 2. The acquisition of datasets and the model training are in Section 3. Scale experiments are in Section 4. Conclusions and future work are in Section 5.

## 2. Indoor Fire Detection Method

**Figure 1:**
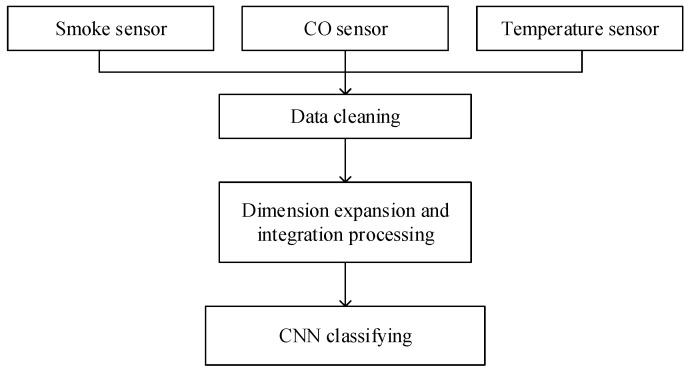
System structure of the indoor fire detection method.

The structure of the indoor fire detection system is shown in Figure 1. Smoke, CO, and temperature sensors convert smoke concentration, CO concentration, and temperature in the environment into electrical signals and obtain corresponding environmental data through analog-to-digital conversion. Then, the environment data are cleaned by an SG filter to avoid the influence on the algorithm when the sensors are disturbed. The GAF method is used to convert time series data of the output of three kinds of sensors into three two-dimensional matrices, which will be expanded and integrated into a three-dimensional matrix. Finally, ConvNeXt-FiRe is used to classify and output the results of fire detection.

### 2.1. Sensor Signal Pre-Processing

In order to convert time-series data of the output of three kinds of heterogeneous sensors into a three-dimensional matrix, which could be classified by the CNN to obtain the results of fire detection, we use the SG filter and the GAF method to pre-process the heterogeneous sensor data.

The SG filter [21] is used to process the sensor data, eliminating the fluctuation of the sensor signal caused by noise, and removing the occasional outlier fault caused by interference.

The GAF method is used to convert one-dimensional time series sensor signals into a two-dimensional matrix. The mathematical representation of this approach can be explained as follows [22]:

Firstly, minimum–maximum standardization is used to standardize the data. The formula is as follows:

(1) $$x_{i}^{\prime }=\frac{x_{i}-\max(X)+x_{i}-\min(X)}{\max(X)-\min(X)}$$

where $x_{i}$ is the output value of the sensor at time i. X is time series vector of the sensor, $X=[x_{1},x_{2},x_{3},\cdots x_{n}]$. X is rescaled so that all values fall in the interval [−1, 1]. Then, the standardized data are mapped to the polar coordinate system, where the value is used as the cosine value of the polar coordinate angle and the timestamp is used as the polar coordinate radius. The transformation formula is as follows:

(2) $$\left\{\begin{array}{cc} \phi =\arccos\left({x^{\prime }}_{i}\right), & -1\leq {x^{\prime }}_{i}\leq 1,\;{x^{\prime }}_{i}\in X^{\prime }\\ r=\frac{i}{N}, & 1\leq i\leq N \end{array}\right.$$

where i is the time number of the sequence, the total length of the sequence is N, and $X^{\prime }$ is the rescaled time series. The standardized data range is [−1, 1], and the data after the inverse cosine function transformation satisfy ϕ∈[0,π], r∈[0,1]. Let ${x^{\prime }}_{i}$ and ${x^{\prime }}_{j}$ be two vectors and define the formula:

(3) $${{\;x}^{\prime }}_{i}⊕{x^{\prime }}_{j}=\cos(\phi_{i}+\phi_{j})$$

(4) $$={x^{\prime }}_{i}\cdot {x^{\prime }}_{j}-\sqrt{1-{{x^{\prime }}_{i}}^{2}}\cdot \sqrt{1-{{x^{\prime }}_{j}}^{2}}$$

where $\phi_{i},\phi_{j}$ are the angles of ${x^{\prime }}_{i}$ and ${x^{\prime }}_{j}$, and ${x^{\prime }}_{i}⊕{x^{\prime }}_{j}$ is the correlation between ${x^{\prime }}_{i}$ and ${x^{\prime }}_{j}$. Therefore, the GAF matrix is

(5) $$G_{n\times n}=\left(\begin{array}{cccc} {x^{\prime }}_{1}⊕{x^{\prime }}_{1} & {x^{\prime }}_{1}⊕{x^{\prime }}_{2} & \cdots & {x^{\prime }}_{1}⊕{x^{\prime }}_{n}\\ {x^{\prime }}_{2}⊕{x^{\prime }}_{1} & {x^{\prime }}_{2}⊕{x^{\prime }}_{2} & \cdots & {x^{\prime }}_{2}⊕{x^{\prime }}_{n}\\ \vdots & \vdots & \ddots & \vdots \\ {x^{\prime }}_{n}⊕{x^{\prime }}_{1} & {x^{\prime }}_{n}⊕{x^{\prime }}_{2} & \cdots & {x^{\prime }}_{n}⊕{x^{\prime }}_{n} \end{array}\right)$$

The GAF method provides a way to preserve temporal dependency, since time increases as the position moves from the top left to the bottom right. It converts a time series of length *n* to the n×n GAF matrix, and converts each point of the time-series data into a correlation between that point and other points. The GAF matrix contains temporal correlations, since $G_{\left(i,j|i-j|=k\right)}$ represents the relative correlation by superposition in the direction of time interval k. The main diagonal $G_{i,i}$ is a special case when k=0, which contains the original angular information. Therefore, the GAF matrix enlarges the characteristics of time-series information.

The GAF method is then used to transform the three fire signal sequences into three matrices and aggregate the three matrices as three channels into a three-dimensional matrix. Finally, the CNN is used to detect potential cases of fire.

### 2.2. ConvNeXt Network Model

Liu et al. [20] listed five different structural models of ConvNeXt from small to large. Among them, the ConvNeXt-T model is the one with the least amount of calculation, although it is still too large for the practical embedded fire detection devices.

Thanks to the pre-processing stage, the characteristics of time-series information are better represented. Thus, we can further decrease the blocks, channels and layers of the ConvNeXt-T model. Specifically, we keep four stages, like those of [20], but decrease (1) the number of blocks in each stage from (3, 3, 9, 3) in ConvNeXt-T to (1, 1, 3, 1); (2) the number of input channels in each stage from (96, 192, 384, 768) in ConvNeXt-T to (24, 48, 96, 192); and (3) the expansion ratio of the middle hidden layer of the block from 4 in ConvNeXt-T to 2. These reduce the number of convolution calculations so that the computational complexity is greatly decreased.

**Figure 2:**
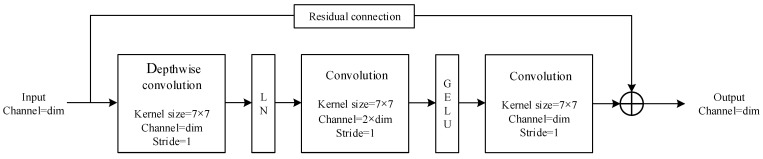
The block structure of the ConvNeXt-FiRe model.

**Figure 3:**
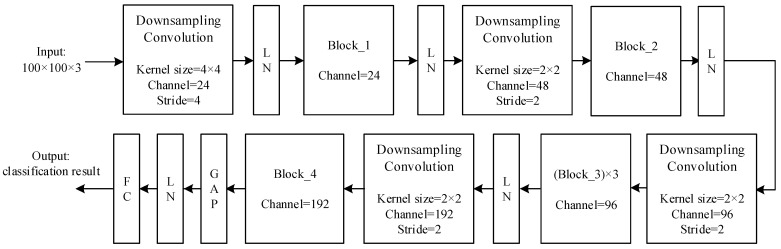
The overall structure of the ConvNeXt-FiRe model.

The block and overall structure of the network, named ConvNeXt-FiRe, are shown in Figure 2 and Figure 3. GAP is a global average pooling layer and FC is a fully connected layer.

## 3. Dataset Acquisition and Model Training

### 3.1. Acquisition of Datasets

The datasets of this experiment are divided into two categories: positive samples and negative samples.

The positive samples describe the fire scenarios. To gather a substantial amount of fire scenario datasets, Fire Dynamic Simulator (FDS) [23], which is a well-known fire dynamic simulation method, is used here. Thunderhead Engineering PyroSim (PyroSim), a three-dimensional visual modeling tool based on this method, can model and simulate the fire numerical values of indoor scenes, accurately simulate the evolution of indoor fires, and record the changes in various sensor data at various locations to obtain datasets. The negative samples are datasets in non-fire scenarios, which are collected by three sensors in the indoor natural environment and interference environment.

#### 3.1.1. Acquisition of Positive Samples

(1) Modeling with PyroSim

In order to carry out the simulation experiment of combustion, a scene is modeled in PyroSim according to the scale experiment conditions. The grid parameters of the modeling scene are set as shown in Table 1.

**Figure 4:**
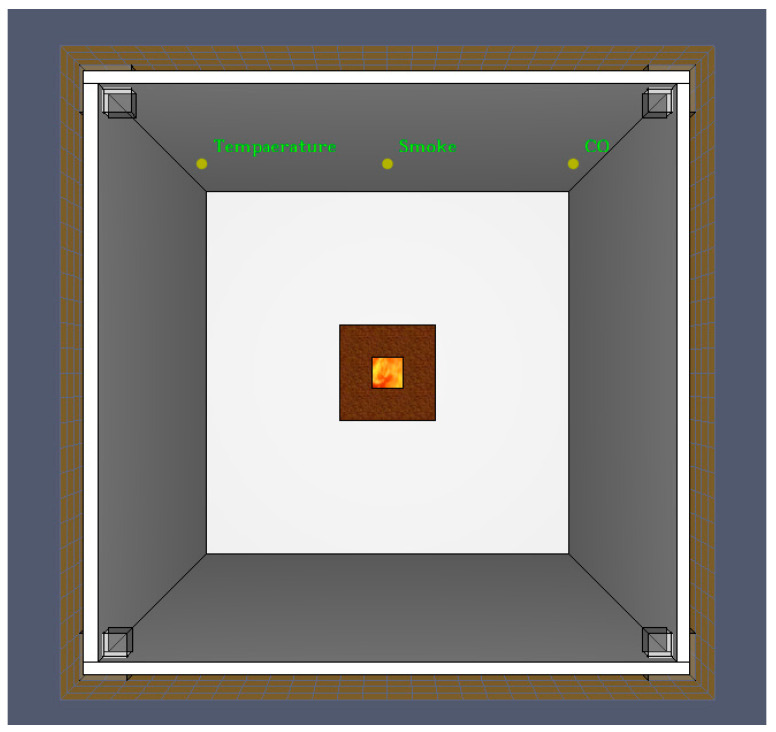
The position of the sensors and fire source.

The position of the three sensors is consistent with that of the scale experiment and the arrangement interval is 0.8 m. The fire source is T square type and its heat release rate is 1055 kW/m^2^. The size of the fire source is 0.2 m × 0.2 m and the power of it is 42.2 kW. The position of sensors and fire sources in the modeling scene are shown in Figure 4.

(2) Numerical Simulation of Indoor Fire

**Figure 5:**
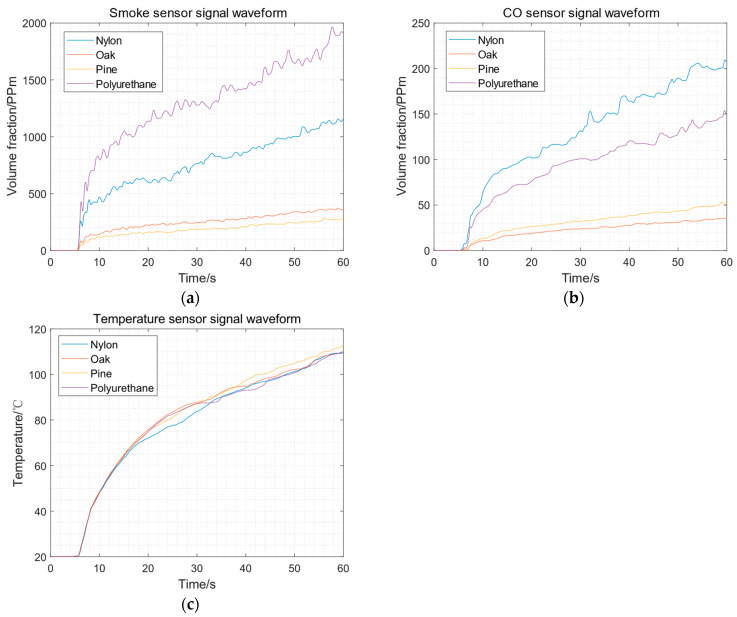
The results of fire numerical simulation: (**a**) smoke sensor signal waveform; (**b**) CO sensor signal waveform; (**c**) temperature sensor signal waveform.

The environmental parameters of fire numerical simulation are as follows: the temperature is 20 degrees Celsius, the relative air humidity is 40%, the air pressure is 1.0132 × 10^5^ Pa, and the oxygen mass fraction is 0.2324. There are four kinds of burners: nylon, oak, pine, and polyurethane. Each experiment lasted for 60 s, and six groups of positive samples could be obtained. Each type of burner was simulated 96 times to obtain ample positive samples. The signals of the sensors with four kinds of burners are shown in Figure 5. The CO and smoke data are calibrated in parts per million of volume fraction, and temperature data are measured in degrees Celsius.

#### 3.1.2. Acquisition of Negative Samples

**Figure 6:**
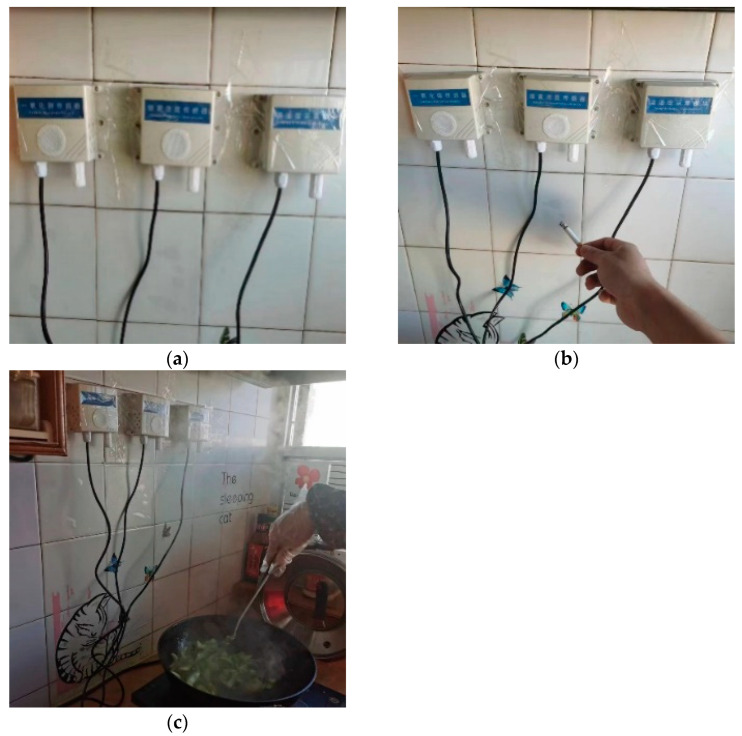
The collection environment of negative samples: (**a**) indoor natural environment; (**b**) indoor environment with cigarette interference; (**c**) indoor environment with oil smoke interference.

The collection environment of the negative samples is shown in Figure 6. The sampling period of the sensor is 0.1 s, so the length of the data sampled in the 10 s time window is 100. The units of the three types of data are consistent with the positive samples.

The sampling interval of the positive sample is 0.0787 s. In order to maintain consistency with the sampling frequency of the real sensor, the positive sample is downsampled in time. The number of sampling points of positive samples in each time window is 166 points, which is integrated into 100 points by resampling. The number of positive and negative samples is 2286, respectively. Datasets are divided into a training set and a validation set according to a ratio of 4:1 for training the network model.

### 3.2. Multi-Heterogeneous Sensor Signal Pre-Processing

**Figure 7:**
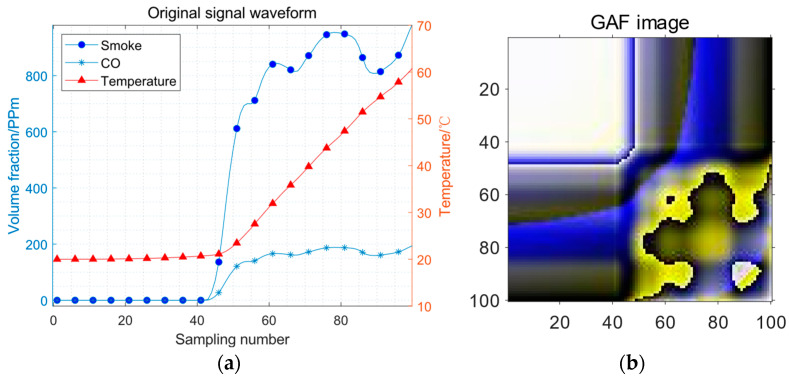
The GAF image of positive samples: (**a**) the original signal waveform; (**b**) the GAF image.

The data from the three sensors are cleaned by means of the SG filter, eliminating the fluctuation of the sensor signal caused by noise, and removing the occasional outlier fault caused by interference. The GAF method is then used to transform the cleaned data into three matrices and aggregate the three matrices as three channels into a three-dimensional matrix. The result of the positive samples is shown in Figure 7.

**Figure 8:**
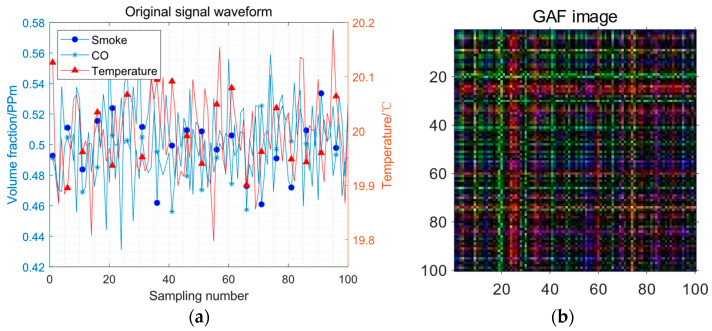
The GAF image without fire: (**a**) the original signal waveform; (**b**) the GAF image.

When there is no fire, the original signal of the fire sensor under the influence of environmental noise and the result of negative samples is shown in Figure 8.

Figure 7a and Figure 8a adopt double coordinate axes, in which the volume fraction of smoke and CO adopts the left coordinate axis, and the temperature adopts the right coordinate axis. Figure 7b and Figure 8b as obtained by the GAF method, are square images with a sequence length.

### 3.3. Model Training

After pre-processing, the time series in the detection time window will be transformed into a 100 × 100 sensor feature image, so the size of the network input feature image in this paper is designed to be 100 × 100. The Adam optimizer is used to train the ConvNeXt-FiRe model. The hyperparameters are set as follows: the training round is 9, the sample batch size is 8, the initial learning rate is 5 × 10^−4^, and the weight attenuation coefficient of the optimizer is 5 × 10^−2^. Since it is a binary classification task, the cross entropy function is used as the loss function for training. The formula is as follows:

(6) $$L=\frac{1}{N}\sum_{i}Li=-\frac{1}{N}\sum_{i}\sum_{c=1}^{M}y_{ic}\log(p_{ic})$$

where *M* is the number of classification categories.$y_{ic}$ is a sign function. If the real class of sample i is equal to c, its value is 1; otherwise, it is 0. $p_{ic}$ is the prediction probability that the observed sample i belongs to class c.

The number of positive and negative samples is 2286, respectively. Datasets are divided into a training set and a validation set according to a ratio of 4:1 for training the network model.

**Figure 9:**
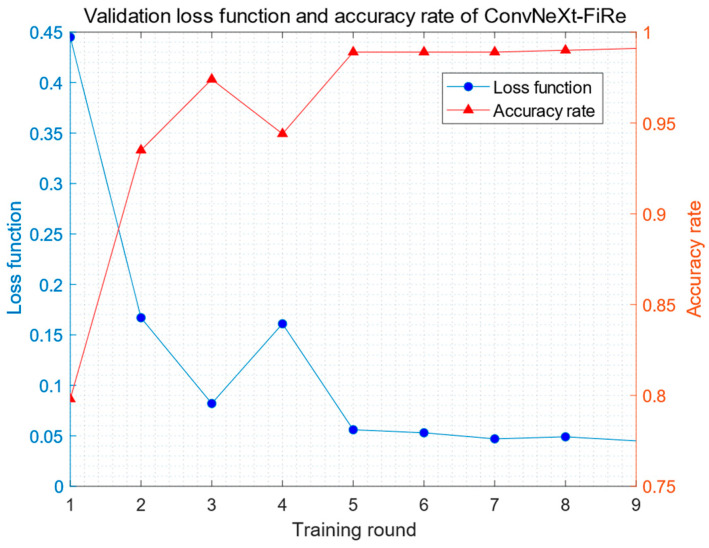
The results of training the ConvNeXt-FiRe model.

The result of training the ConvNeXt-FiRe model is shown in Figure 9. After nine rounds of training, its classification accuracy reached 99.1%.

In order to avoid the impact of model size inconsistency on performance, the computational load of BPNN and LSTM models is designed to be consistent with that of the ConvNeXt-FiRe model in the method comparison experiment, while keeping the training parameters consistent.

Table 2 presents the performance comparison between different models. It can be seen that the number of parameters of the LSTM model is the smallest, but the accuracy is the lowest. The number of parameters of the BPNN model is the biggest, but the accuracy is nearly the same as that of the LSTM model. The ConvNeXt-FiRe model’s number of parameters is slightly bigger than that of the LSTM model, while the accuracy is the highest. Therefore, the ConvNeXt-FiRe model can be better applied to the resource-constrained embedded platform of an indoor fire detection system.

## 4. Experimental Verification

**Figure 10:**
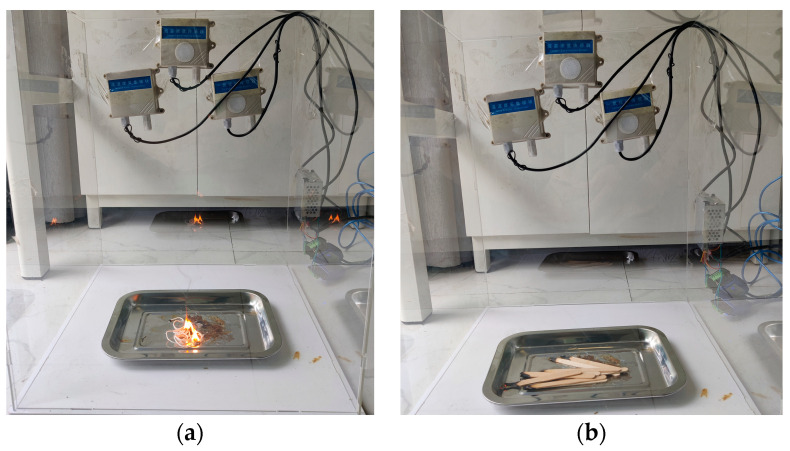
The scene of the scale experiment: (**a**) cotton thread combustion experiment; (**b**) wood smoldering experiment.

Due to the high risk of strong pollution and the high cost of a real fire experiment, it is difficult to implement in an actual indoor scenario. In this paper, a scale experiment in a semi-closed environment is designed. Specifically, the scale experiment is carried out on a scale of 0.4 m × 0.4 m × 0.5 m acrylic cube space at a ratio of 1:6 to simulate the occurrence of fire, so as to verify the performance of the method on an embedded platform. The experiment scene is shown in Figure 10.

In the scale experiment, two scenarios of cotton rope combustion and wood smoldering fire were selected to simulate the combustion of furniture, clothing and other items when an indoor fire breaks out, and combustion was carried out in a metal container. The alarm signal of the detection system is output to the server through the network interface.

**Figure 11:**
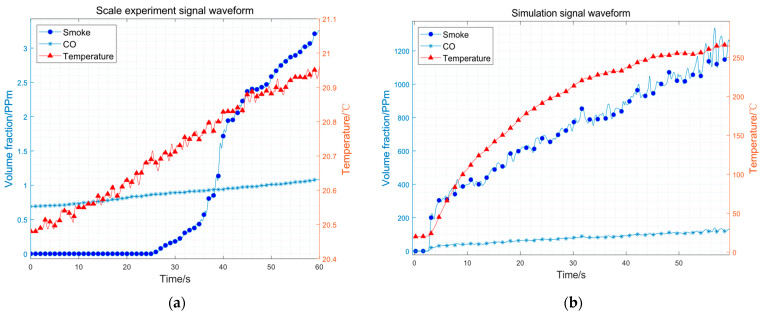
Sensor signal waveform of (**a**) the scale experiment, (**b**) the simulation.

Figure 11 illustrates the data captured by three sensors during the wood smoldering scale experiment and the simulation utilizing oak as the burning material. Notably, the data from all three sensors exhibit a consistent upward trend over time in both the scale experiment and the simulation. However, due to the limitations of the scale experiment, the values of the sensor data are quite different. Thanks to the minimum–maximum standardization in the GAF method, the scale experiment data can be correctly classified, although they are quite different from those of the simulation.

The detection system’s classification results of the wood smoldering scale experiment are shown in Table 3. The sampling period of the sensor is 0.1 s, so the length of the data sampled in the 10 s time window is 100. There are six time periods in 60 s. Within 0~20 s, the smoke data remain relatively close to 0. Thus, the non-fire probability is 0, and the classification result is non-fire. Within 20~30 s, the smoke data begin to increase in the latter half. Thus, the non-fire probability changes to 0.883, and the classification result is still non-fire. Within 30~60 s, all three data sensors show an upward trend, aligning with the positive sample of the training datasets. Thus, fire probability is higher than non-fire probability, and the classification result is fire. The scale experiments effectively verified the feasibility of employing the method in a resource-constrained embedded platform of an indoor fire detection system.

## 5. Conclusions

Indoor fires pose significant threats in terms of casualties and economic losses globally. Aiming to combat the shortcomings of existing indoor fire detection methods, this paper proposes an indoor fire detection method based on multi-sensor data fusion and a lightweight CNN. By aggregating and expanding the three types of heterogeneous sensor data, the time-series data are transformed into a three-dimensional matrix format similar to the picture, facilitating fire detection by using the CNN for image classification. With the help of numerical simulation experiments, the feasibility of data pre-processing and CNN classification in detecting fire is verified. Compared with the existing methods, the accuracy of the proposed method is higher, while the number of CNN parameters and the amount of computation are still small, which means the proposed method is more suitable for the embedded platform of an indoor fire detection system. Subsequent scale experiments verified the feasibility of employing the method in a resource-constrained embedded platform of an indoor fire detection system.

## Figures and Tables

**Table 1:** Modeling grid parameters.

Actual Space Size	Modeling Space Size	Grid Number
2.4 m × 2.4 m × 3 m	2.6 m × 2.6 m × 3.2 m	20,280

**Table 2:** Performance comparison between different models.

Model Name	Parameters	FLOPs	Accuracy
BPNN [15]	12.4 M	12.41 M	93.40%
LSTM [19]	218.88 K	12.27 M	92.90%
ConvNeXt-FiRe	399.91 k	12.58 M	99.10%

**Table 3:** The wood smoldering scale experiment classification results.

Time Period	Non-Fire Probability	Fire Probability	Classification Result
0~10 s	1.0	0	Non-fire
10~20 s	1.0	0	Non-fire
20~30 s	0.783	0.217	Non-fire
30~40 s	0.294	0.706	Fire
40~50 s	0.246	0.754	Fire
50~60 s	0.341	0.659	Fire

## Data Availability

No new data were created or analyzed in this study. Data sharing is not applicable to this article.
